# A methodology for measuring the form of organic settlements

**DOI:** 10.1016/j.mex.2019.02.009

**Published:** 2019-02-15

**Authors:** Egin Zeka, Mehmet Ali Yuzer

**Affiliations:** a Graduate School of Science, Engineering, and Technology, Istanbul Technical University, ITU Ayazaga Kampusu, 34496, Maslak, Istanbul, Turkey; b Department of Urban and Regional Planning, Faculty of Architecture, Istanbul Technical University, Harbiye Mahallesi, Taskisla Cad., 34367, Sisli, Istanbul, Turkey

**Keywords:** Organic urban form measurement, Urban morphology, Measuring urban form, Organic settlement, ArcGIS

## Abstract

This article introduces a methodology that aims to develop a detailed framework and guideline for analyzing and measuring the urban form of any settlement, and especially the urban fabric that represents an organic layout. The basis of the methodology lays on Conzenian approach that analyzes the urban morphology by categorizing it in three main elements: streets, plots, and buildings. Since then, many studies are conducted in the field of urban morphology but only few of them focuses on measuring the spatial features of the urban space. Especially, the measurement of irregular (organic) settlements has been a real challenge since it represents a high complexity that is difficult to be measured with mathematical tools. This study tries to develop a method that is useful for measuring the spatial qualities of an organic historic settlement. Firstly, it makes a more detailed categorization and structure of the urban components. Secondly, it makes detailed mathematical measurement to extract the distances, dimensions, areas, proportions and other relationships in the settlement in different scales. The methodology uses AutoCAD and GIS tools for producing maps and making different measurements.

• The methodology is useful for measuring the complexity of urban form in organic settlements.
• It makes a detailed framework of urban components and is helpful for synthesizing the information and understanding the relationships among different units of the complex urban system.
• It can be useful to develop an automated software/tool that makes these measurements and makes correlations between different features of measured units.

## Graphical abstract

**Figure:**
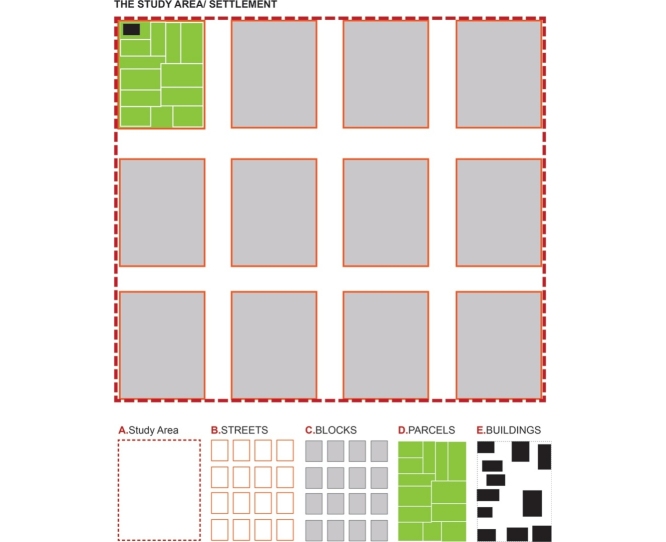

Specifications table

**Table:** 

Subject Area	• Engineering • Environmental Science
**More specific subject area:**	Urban Studies, Urban Morphology
**Method name:**	Organic Urban Form Measurement
**Name and reference of original method**	
**Resource availability**	

## Method details

### Basis of the concept

The study of urban form has been in the focus of many scholars for a long time but as scientific discipline it dates back to the first part of 20th century [1]. From that time, various approaches are developed in the field of urban morphology. The methodology of this article is based on the approach of M.R.G Conzen, one of the pioneers in the urban morphological studies, who make a clear and simple definition of urban form [2]. According to him, the urban form consists of three basic components: the streets; the parcels; and the buildings [3]. The footprint of streets, parcels and buildings define the city in two-dimensional aspect, while, the building fabric is important for the third dimension and the function it provides [1,4,5]. A similar approach is developed by Jason who lists three morphological units as the basic ones; the plan of the streets; the building form; and the function of both [6].

**Fig. 1:**
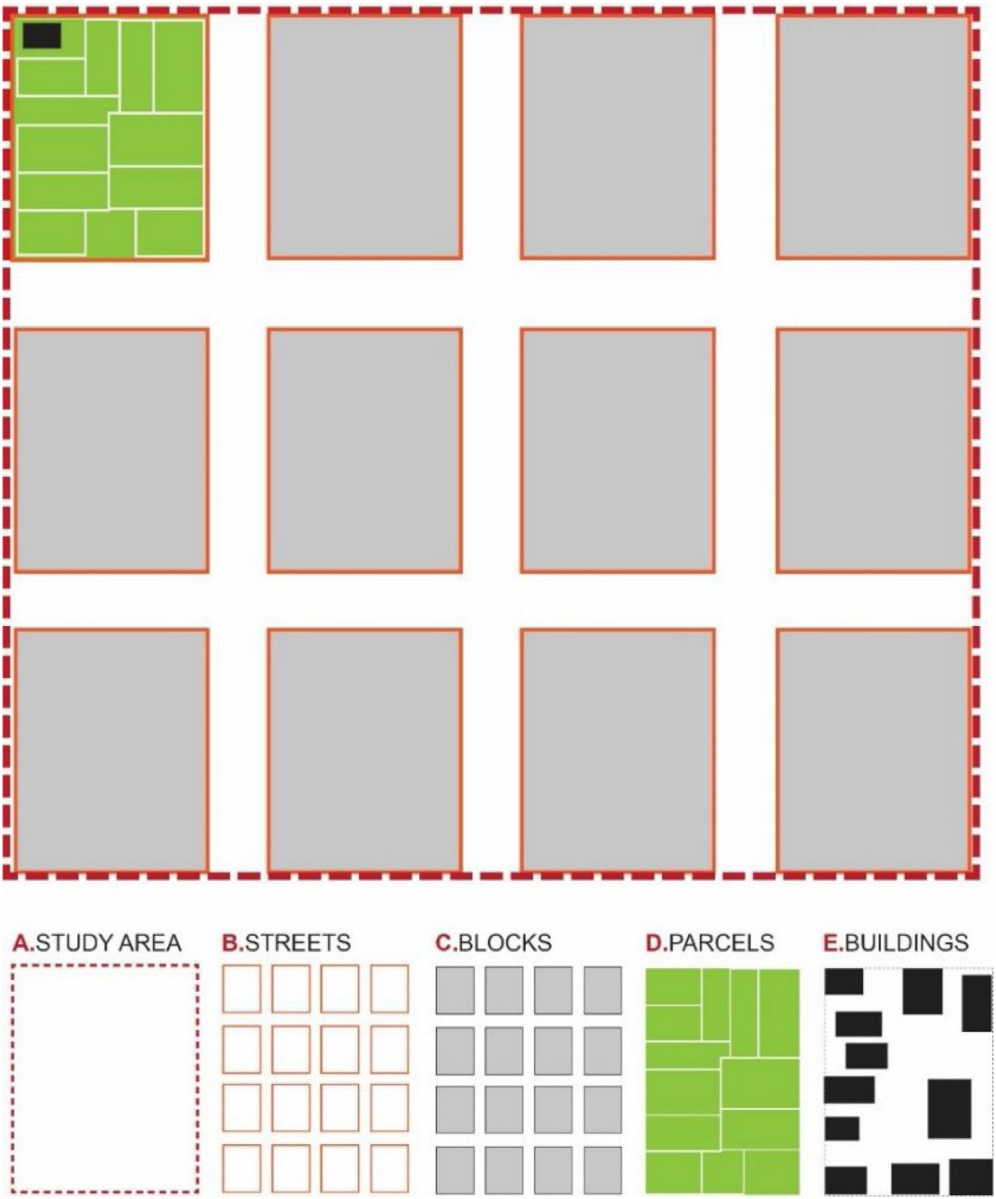
Diagram showing the morphological units.

**Fig. 2:**
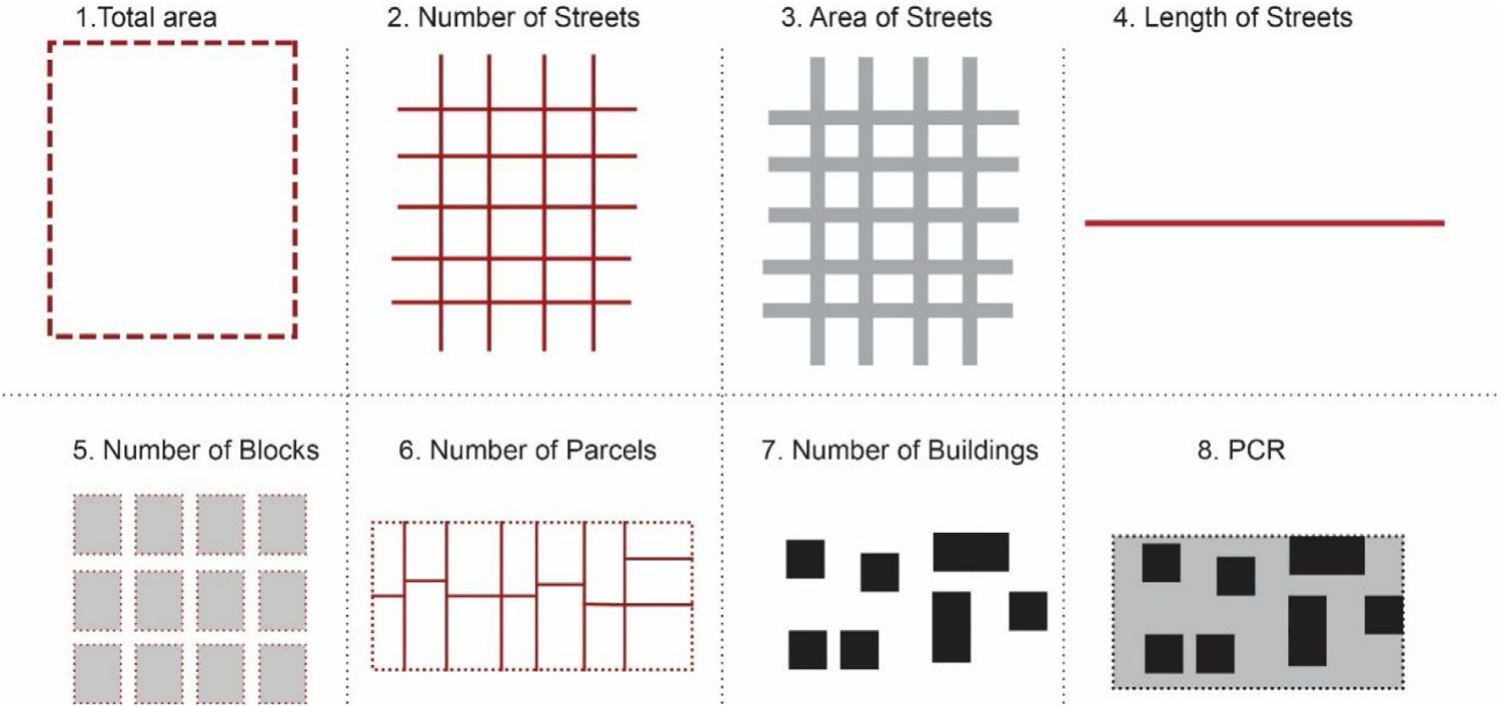
Parameters to be measured in overall study area/settlement.

The methodology presented here follows the concept of Conzen by adding a new morphological component which is ‘the block^1^’ and more specifically it develops an expanded structure of 32 subcategories and parameters in total [Table 1]. It starts with some analyses and measurements of general characteristics of the study area/settlement. Further, it develops a methodology for analyzing four main morphological units: the streets; the blocks; the parcels; and the buildings [Fig. 1]. A detailed analysis of different aspects of each morphological unit contributes in the understanding of their characteristics. The procedure proposes various parameters to be measured and produces quantitative information for each of them in different scales. These measurements provide useful information such as: distances, areas, proportions, frequencies etc (Fig. 2).

**Table 1:** The detailed structure of main morphological units, criteria and measurement parameters used in this methodology.

		Number value	Area	Distances	Proportions	Dimensions	Orientation	Angle
	**Criteria**							
**A**	**Study area/District**							
1	Total area		x					
2	Total Number of Streets	x						
3	Total Area of Streets		x					
4	Total Length of Streets			x				
5	Total Number of Blocks	x						
6	Total Number of Parcels	x						
7	Total Number of buildings	x						
8	Plot Coverage Ratio (PCR) ^1^	x			x			
**B**	**Streets**							
1	Orientation of streets						x	x
2	Angular position							
3	Length between intersections			x				
4	Street width			x				
5	Width change frequency	x						
6	Width shift dimensions			x		x		
7	Proportions: street width/building height				x			
8	Directional changes angle							x
9	Street edge			x				
**C**	**Blocks**							
1	Area of blocks		x					
2	No. of parcels/Block	x						
3	No. of buildings/Block	x						
4	Plot coverage area /Block	x			x			
5	Block Dimensions							
**D**	**Parcels/plots**							
1	Area		x					
2	Dimensions					x		
3	Street orientation						x	
4	Proportions				x			
**E**	**Buildings**							
1	Location on parcel						x	
2	Orientation						x	
3	Setbacks			x				
4	Buildings along the street							
5	Solid void relationship							
6	Vertical rhythm				x

This method contributes to urban morphology studies in different aspects. Firstly, it makes a clear structure for the procedure of urban form analysis, as well as it develops a step-by step structure by detailing the criteria and categories in a chronological and hierarchical way. Secondly, it suggests the types of measurements that can be done in morphological analyses in order to extract different features of the settlement. These measurements are important not only for providing quantitative information, but also for their contribution in understanding the primary principles that shape the urban space. The statistical data extracted through this study shows the main tendencies of different compositions. The revealed quantitative data can be used to make correlations and synthesis about the relationship of different parameters. Another contribution of this method is that it builds a detailed framework for future studies that may focus on developing a software tool that can make the measurements in an automated process. Finally, the methodology proposed here can assist in developing models for new settlements that are in harmony with existing urban fabric, especially in historic urban contexts. The information that can be synthesized from these morphological analyses can be easily incorporated in any model that tries to generate similar principles in the composition of the urban space.

### The procedure

The methodology consists of several steps that are organized in an incremental process. The first step is the provision of the data that represents the study area in maps in a vectoral format. The maps are processed in AutoCAD and ArcGIS programs for making different data organization and measurement. Firstly, the information of morphological elements is categorized in five different layers in AutoCAD: the study area boundary; the street lines; the block lines; the parcel lines; and the building lines. Next, each of these layers is converted in polyline and polygon format in AutoCAD. The following procedure makes detailed calculations using AutoCAD and ArcGIS software for all the parameters presented in Table 1. After that the data of each parameter is imported in Excel to make data organization and to understand the distribution or frequencies of different features. Finally, a data set representing the profile of each morphological unit, in aspect of the quantitative features that it poses, is prepared.

### Calculating the general characteristics of the study area

The first step of the procedure is to define the study area and its core features regarding the main components of urban form such as: the area of the study zone; area of streets; number of blocks; number of parcels; number of buildings; and plot coverage area. These parameters provide a general view that helps to understand how the settlement is subdivided in smaller morphological units. The technical procedure is conducted both in AutoCAD and ArcGIS. Firstly, as mentioned above, all the components are categorized in layers and all of them are converted in polyline and polygon format in AutoCAD. After that, the layers are exported to ArcGIS separately. Each of the layers is converted in shape files in ArcGIS.

The calculation of the surfaces is done in following steps in ArcGIS: Select the component > open attribute table > table options > add new filed > Name = surface, Type = long Integer > Ok > the surface column is created > right click on the column > calculate geometry > property = area, units = m or ha > OK> the area of the selected unit is calculated. This procedure is similar for calculating total study area surface; the surface of streets; the surface of blocks and the surface of buildings.

To calculate the total number of any morphological unit: select the component > open attribute table > the total number is given at the end of the table. All the numerical data generated in ArcGIS can be exported to excel for their further analyses. The procedure for this is: Open the attribute table of the component > table options > select all > right click on the table > copy selected > open a new excel sheet > paste the data. For example, to calculate the Plot Coverage Ratio in overall, both the data of block surfaces and building surfaces are exported to excel and then divided to each other to provide the surface ratio.

To calculate the length of a polyline, in this situation the length of the street segments, the steps are: select the street polyline layer > open attribute table > table options > add new filed > Name = Length, Type = Double > Ok > the length column is created > right click on the column > calculate geometry > property = length, units = m or km > OK> the length of the selected unit is calculated.

### Street pattern measurements

**Fig. 3:**
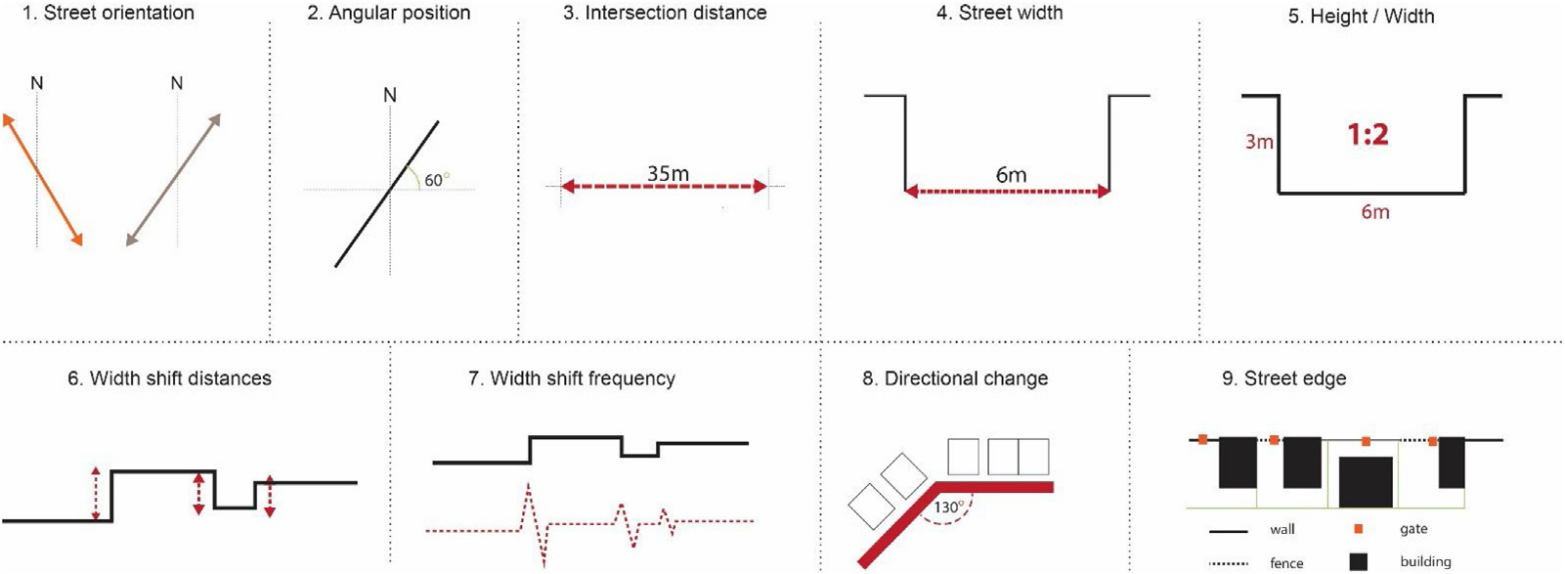
Different parameters that are used to measure the features of the street pattern.

The analyzing process of the street features is very crucial since the street is a core morphological unit defining the form of the settlement. In this methodology nine different measurements are done to understand various characteristics of street pattern and make synthesis and correlations of the calculated information [Fig. 3]. For example, the orientation of the streets is an important information that is related to environmental aspects like sun orientation or wind direction. In order to have this information the orientation of each street segment is defined by calculating its position in the coordinate system. The procedure is done in AutoCAD by intersecting each segment with a vertical line (north south direction) and a horizontal line (east-west direction). After that the angle of the street orientation is calculated by the angle command. Another feature that is related to street orientation is the directional change of the street in any particular angle. This is helpful to understand the dynamism of the movement in a settlement. For example, in grid structures almost all of the streets are intersected at perpendicular angles creating a homogeneous and monotonous movement type. However, the organic settlements represent a different character where the streets are commonly intersected in wide angels (between 90° and 180°) by creating a dynamic movement and spatial composition. Calculating the directional changes should be an important measurement to understand the quantitative aspect of this dynamism. To do this, two adjacent street segment are selected > type angle command > the angel is provided. All the numerical data provided by these measurements are put in excel to have further analyses and correlations^1^. By generating charts in excel we can understand the distribution of the measured data such as ‘the most’ and ‘the less’ common directions or angles that are present in the settlement. This procedure can help to understand the common trends on street orientation and directional changes.

**Fig. 4:**
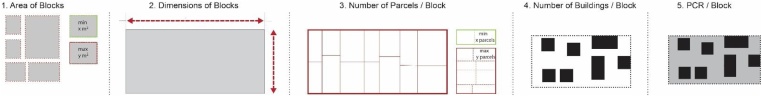
Different parameters that are used to measure the features of the block pattern.

**Fig. 5:**
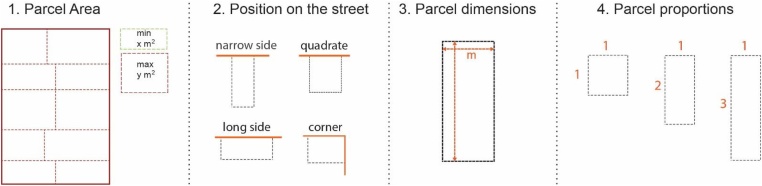
Different parameters that are used to identify the features of the parcels.

**Fig. 6:**
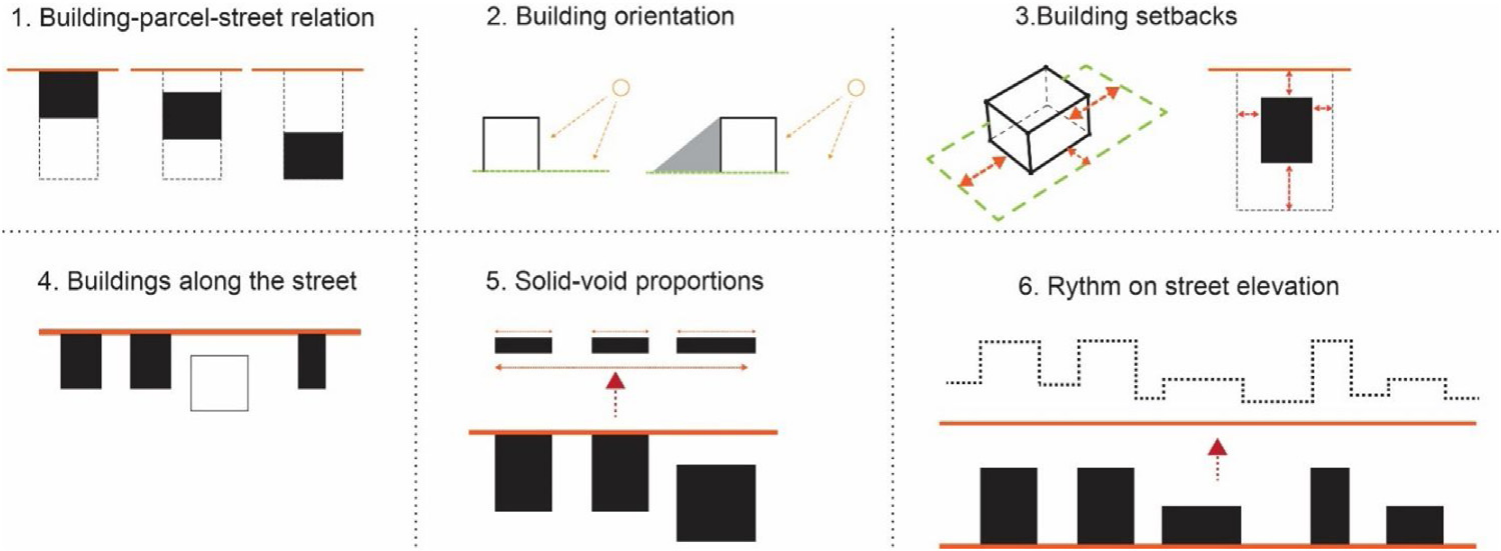
Different criteria that are used to understand the features of building fabric.

Another significant step represented in this method is the measurement of different parameters related to street width such as: street width; width/height ratios; width shift distances; and width shift frequency [Fig. 4, Fig. 5, Fig. 6, 7]. This measurement is conducted in various part of the settlement with the purpose of understanding the common street width and its differences. The proportion of building height to street width identifies the enclosed character of the street in third dimension. This feature is measured in parts where the street edge is enclosed by building facades. The streets in organic settlement, different from geometric urban areas defined by straight lines, are framed by shifting of the line that defines the street border. To detect the features of this complex phenomenon, many measurements should be done to extract two types of information: the frequency of the changes and the distance of width change. This procedure can help to export and analyze data that shows the quantitative aspect of this feature in organic urban fabric.

### Block pattern measurement

The block is a morphological unit positioned between public space (streets) and private zones (parcels and buildings). The outer part of the block consist of street line and the inner part is formed by parcels and buildings. The characteristics of block pattern are measured through the steps shown in Fig. 4. The first step consists of identifying the surface for each block. This is done in a similar procedure shown above for calculating the areas in ArcGIS. Then, the numerical data exported to excel is analyzed to get the information such as: the most common surface of a block, the largest, and the smallest block area. Another measurement done in this section is the identification of block dimension. In organic settlements the blocks do not have regular shapes to be measured accurately. A helpful method might be to calculate the longest distance of length and width of the block in two directions. Following these steps, the blocks are analyzed in aspect of their subdividing units that are the parcels and the buildings. In this context, the number of parcels/blocks; the number of buildings/blocks; and the plot coverage ratio are calculated. The procedure of calculating the number of a particular component, and the plot coverage ratio is shown in a previous section of this article. The similar steps can be performed to make the calculation in the block scale. The numerical data processed in excel gives the most common, the minimum, and maximum values for each parameter.

### Parcel pattern measurement

The parcel is considered a very basic morphological unit since its form is very decisive in defining both the street pattern and the building form, and as a result the overall character of the urban fabric. Several surveys and measurements can assist to identify parcel typologies and features [Fig. 5]. Firstly, the surface of each parcel is measured in ArcGIS and analyzed in excel in the similar procedure shown above. The statistical data received here can show important information such as: minimum and maximum parcel surface, and the most common typology related to parcel area. Being a crucial parameter, the area is not sufficient to understand the features of parcel pattern. The parcel dimensions and their proportions are fundamental in identifying their pattern. To do that, the width and depth of all parcels is measured in AutoCAD. The numerical values are categorized in several intervals for both depth and width. All categorized data is imported to excel and is processed to identify features such as minimum/maximum width; min/max depth; and most common interval for both. Further, the proportions between width and depth are calculated and same quantitative charts are generated. A final step related with parcel features is the identification of their location in relation to the street. This analysis identifies four main categories: narrow side; long side; quadrate parcel; and corner parcel. Each typology is counted and generated as a quantitative information.

### Building pattern measurement

The building is the smallest morphological unit to be analyzed but the most important one in defining the form and the function of the city. In this methodology, the buildings are elaborated in different aspect related to their form, orientation, location on parcel, and the spatial relationship they create with each-other. An important observation is the analysis of building location on the parcel and its orientation [Fig. 6 (1,2)]. In this procedure buildings are categorized according to their position on the parcel and the orientation of their front façade [Table 2]. After the calculations, important parameters such as the most common orientation of buildings and the position of the courtyard are extracted.

**Table 2:** Matrix showing the location of buildings on parcel and their orientation.

	N	NE	E	SE	S	SW	W	NW	total
F									
B									
1S									
2S									
FB									
F1S									
F2S									
B1S									
B2S									
FB1S									
FB2S									
NY									
Total									

F: Just front yard.

B: Just back yard.

1S: Yard just on one side.

2S: Yards on both sides.

F1S: Yard on front + one side.

F2S: Yard on front + both sides.

B1S: Yard on back + one side.

B2S: Yard on back + both sides.

FB1S: Yard on back, front and one side.

FB2S: Yard on all sides of the building.

NY: No yard, building cover all the parcel.

N: North.

NE: North-east.

E: East.

SE: South-east.

S: South.

SW: South-west.

W: West.

Another important calculation is the buildings’ setback distances from the parcel line. For each parcel the average distance from all its sides to the parcel border line is measured. The large amount of quantitative data is analyzed in excel to provide a detailed categorization of building setbacks and distances between each other. Other analyses on building fabric include the calculation of buildings that have a façade on the street edge; the proportion of building facades and void space in elevation of the street; and the rhythm of height change along the street elevation. The data provided from these analyses is crucial in developing a detailed quantitative framework of different morphological features of buildings and their surrounding components.
