# Supplementary material for: A methodology for measuring the form of organic settlements
Source: MethodsX. 2019 Feb 15;6:368–76. doi: 10.1016/j.mex.2019.02.009 (PMC6396093; doi:10.1016/j.mex.2019.02.009)
Supplement: Supplementary file 1 [file mmc1.docx]

**Supplementary material *and/or* Additional information:**

To provide e better picture of the validation of this methodology, some information about the study area and partial results of the research employing this methodology are shown in this section. This methodology is part of a morphological study done in a historic neighborhood in the city of Korca, Albania. This settlement represents an organic urban fabric which has been a challenge for making different measurements and providing a quantitative framework for different morphological categories.

**
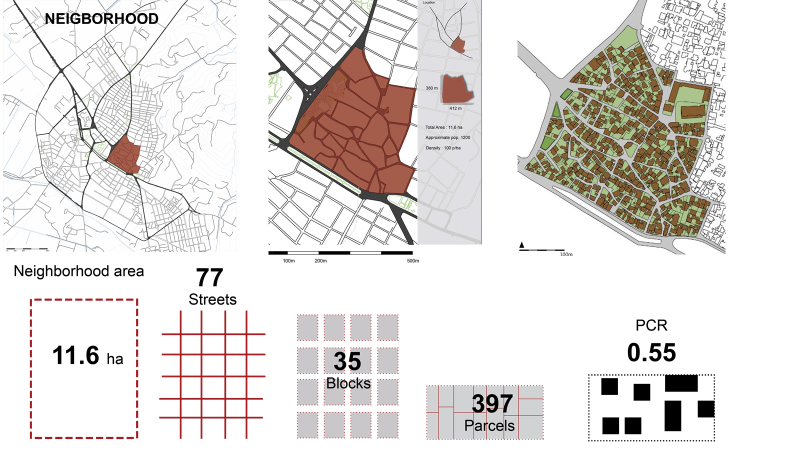
**

Figure 7. The study area. A historic, organic settlement in Korca


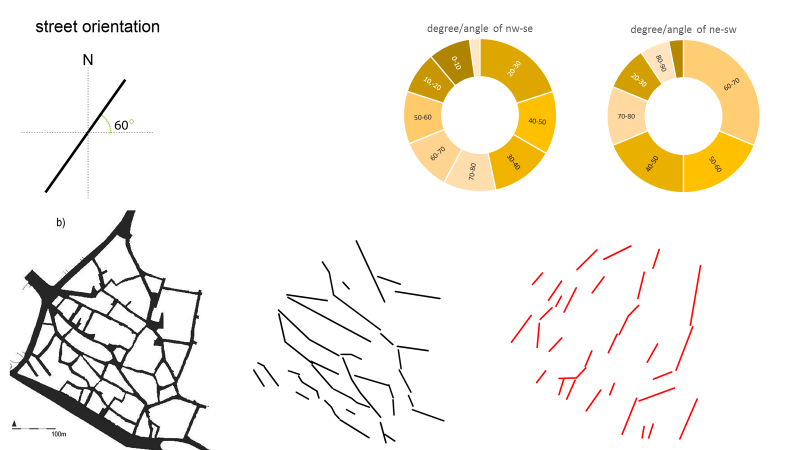


Figure 8. Street orientation and distribution of angles


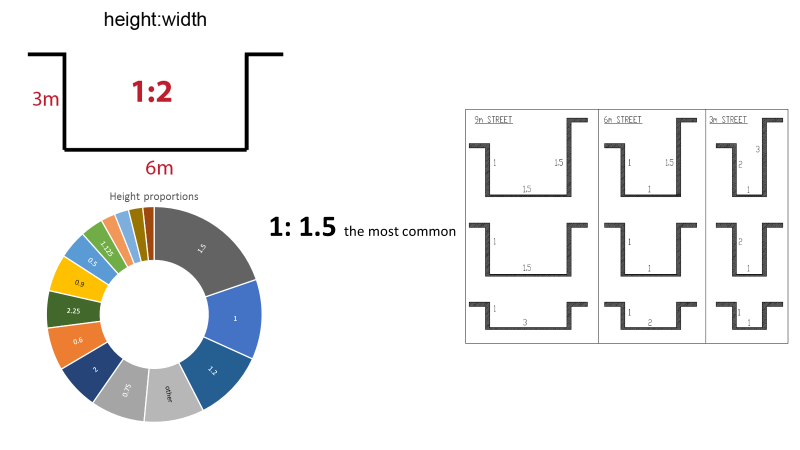


Figure 9. Proportions of building height to the street width


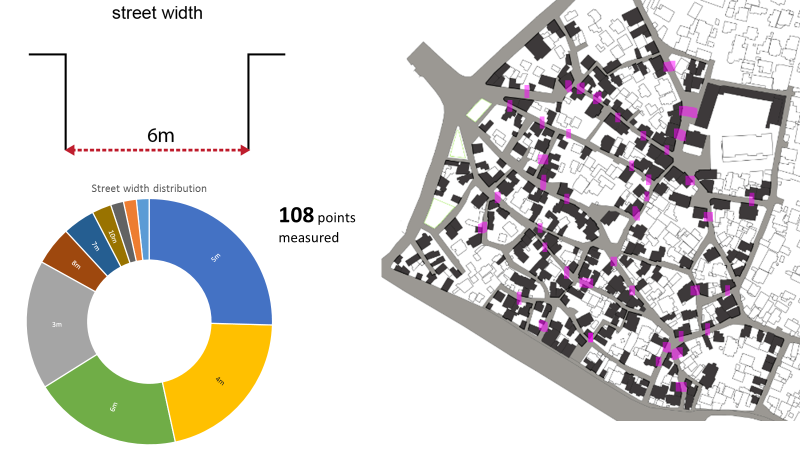


Figure 10. The distribution of street width data


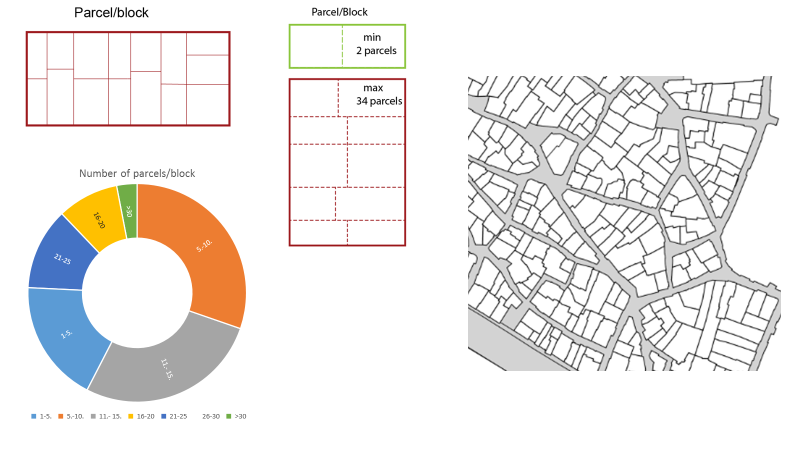


Figure 11. Data about the number of parcels/block


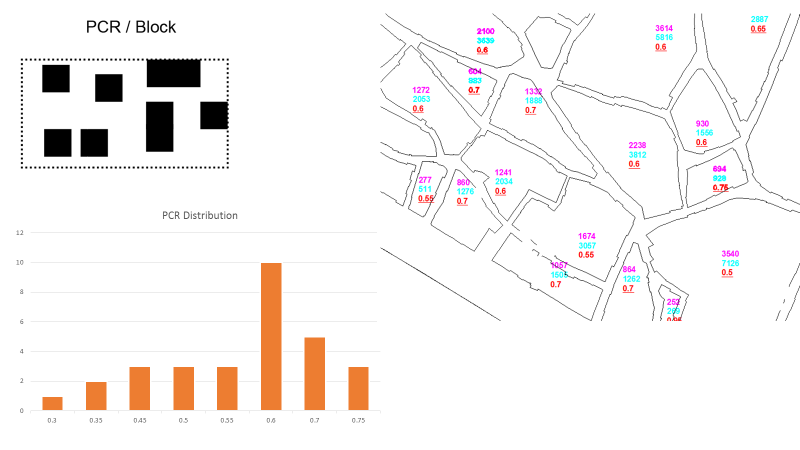


Figure 12. Calculation of plot coverage ratio for each block


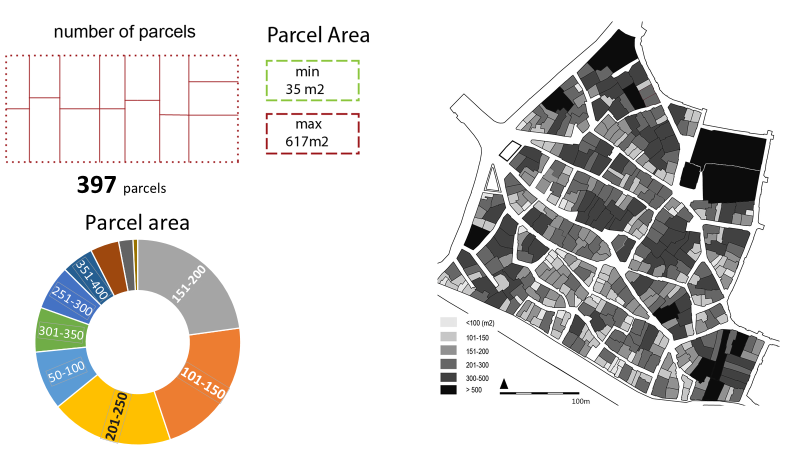


Figure 13. Calculation for parcel surfaces

Figure 14. Location of building on the parcel

Figure 15. Building orientation and yard location

Figure 16. The calculation of building setback distances
